# Enhancement of *in vivo* cardiac photoacoustic signal specificity using spatiotemporal singular value decomposition

**DOI:** 10.1117/1.JBO.26.4.046001

**Published:** 2021-04-19

**Authors:** Rashid Al Mukaddim, Ashley M. Weichmann, Carol C. Mitchell, Tomy Varghese

**Affiliations:** a University of Wisconsin–Madison, Department of ECE, Madison, Wisconsin, United States; b University of Wisconsin–Madison, School of Medicine and Public Health, Department of Medical Physics, Madison, Wisconsin, United States; c Small Animal Imaging and Radiotherapy Facility, UW Carbone Cancer Center, Wisconsin, United States; d University of Wisconsin School of Medicine and Public Health, Department of Medicine/Division of Cardiovascular Medicine, Madison, Wisconsin, United States

**Keywords:** photoacoustic imaging, preclinical murine cardiac photoacoustic imaging, delay-and-sum beamforming, singular value decomposition, spatiotemporal singular value decomposition processing

## Abstract

**Significance:** Photoacoustic imaging (PAI) can be used to infer molecular information about myocardial health non-invasively *in vivo* using optical excitation at ultrasonic spatial resolution. For clinical and preclinical linear array imaging systems, conventional delay-and-sum (DAS) beamforming is typically used. However, DAS cardiac PA images are prone to artifacts such as diffuse quasi-static clutter with temporally varying noise-reducing myocardial signal specificity. Typically, multiple frame averaging schemes are utilized to improve the quality of cardiac PAI, which affects the spatial and temporal resolution and reduces sensitivity to subtle PA signal variation. Furthermore, frame averaging might corrupt myocardial oxygen saturation quantification due to the presence of natural cardiac wall motion. In this paper, a spatiotemporal singular value decomposition (SVD) processing algorithm is proposed to reduce DAS PAI artifacts and subsequent enhancement of myocardial signal specificity.

**Aim:** Demonstrate enhancement of PA signals from myocardial tissue compared to surrounding tissues and blood inside the left-ventricular (LV) chamber using spatiotemporal SVD processing with electrocardiogram (ECG) and respiratory signal (ECG-R) gated *in vivo* murine cardiac PAI.

**Approach:** *In vivo* murine cardiac PAI was performed by collecting single wavelength (850 nm) photoacoustic channel data on eight healthy mice. A three-dimensional (3D) volume of complex PAI data over a cardiac cycle was reconstructed using a custom ECG-R gating algorithm and DAS beamforming. Spatiotemporal SVD was applied on a two-dimensional Casorati matrix generated using the 3D volume of PAI data. The singular value spectrum (SVS) was then filtered to remove contributions from diffuse quasi-static clutter and random noise. Finally, SVD processed beamformed images were derived using filtered SVS and inverse SVD computations.

**Results:** Qualitative comparison with DAS and minimum variance (MV) beamforming shows that SVD processed images had better myocardial signal specificity, contrast, and target detectability. DAS, MV, and SVD images were quantitatively evaluated by calculating contrast ratio (CR), generalized contrast-to-noise ratio (gCNR), and signal-to-noise ratio (SNR). Quantitative evaluations were done at three cardiac time points (during systole, at end-systole (ES), and during diastole) identified from co-registered ultrasound M-Mode image. Mean CR, gCNR, and SNR values of SVD images at ES were 245, 115.15, and 258.17 times higher than DAS images with statistical significance evaluated with one-way analysis of variance.

**Conclusions:** Our results suggest that significantly better-quality images can be realized using spatiotemporal SVD processing for *in vivo* murine cardiac PAI.

## 1 Introduction

Photoacoustic imaging (PAI) is a non-invasive medical imaging modality that couples optical absorption induced molecular contrast with the anatomical contrast of ultrasound (US) imaging at ultrasonic spatial resolution.^1^ Application of PAI has been demonstrated in both clinical (e.g., carotid atherosclerosis,^2^ breast mass differentiation,^3^ melanoma detection,^4^ and cardiac catheter intervention^5^) and pre-clinical settings (e.g., oncology research,^6^ surgical guidance,^7^^–^^9^ and prostate brachytherapy^10^). PAI can also be utilized non-invasively to evaluate blood oxygenation in myocardial tissue,^11^^,^^12^ which can potentially complement existing preclinical (e.g., murine models of ischemia-reperfusion^13^) cardiac imaging methods such US echocardiography, speckle tracking echocardiography,^14^ and cardiac elastography^15^ by providing unique molecular information.

PAI has been used to describe myocardial blood oxygenation information utilizing high persistence (multiple frame averaging) to increase the signal-to-noise ratio (SNR) of myocardial wall PA signals.^11^^,^^16^ This PAI technique leads to reduced sensitivity and resolution (both spatial and temporal) in cardiac photoacoustic (PA) images because of the potential for averaging PA signals from multiple sources [i.e., myocardial tissue, blood in left-ventricular (LV) chamber, and surrounding static muscle tissue] due to the presence of natural cardiac deformation, thus corrupting blood oxygenation quantification in the myocardium. Therefore, avoiding frame averaging is desirable to improve spatial and temporal resolution, and sensitivity to small variations in PA signals from the myocardial wall. However, reconstructed PA images with conventional delay-and-sum (DAS) beamforming^17^ without persistence typically have low SNR. In addition, PA signals from blood inside the LV chamber will also contribute as incoherent clutter signals within the imaging field of view (FOV).^17^ These factors contribute to reduced signal specificity in the myocardial wall rendering cardiac PAI interpretation difficult. Adaptive beamforming algorithms such as spatial and spatiotemporal coherence weighting,^17^^–^^20^ short-lag spatial coherence weighting,^21^ delay-multiply and-sum,^22^ and multiple DAS with Enveloping (multi-DASE)^23^ have been employed to suppress incoherent clutter signals. However, these methods may also undesirably suppress the myocardial wall PA signals during clutter suppression leading to reduced signal specificity. Recently, machine learning-based PA image formation methods have also been reported.^24^^,^^25^ However, adaptation of these methods for murine *in vivo* cardiac PAI requires appropriate training dataset synthesis incorporating complicated cardiac deformation, physiology, and US physics.

In this paper, we report on a spatiotemporal singular value decomposition (SVD) processing method using electrocardiogram and respiratory signal (ECG-R) gating with *in vivo* cardiac murine PAI data beamformed with DAS.^26^ SVD has been previously used for artifact and clutter reduction in US imaging,^27^ power Doppler,^28^^,^^29^ and ultrafast functional US imaging,^29^^–^^31^ demonstrating remarkable improvement in sensitivity. Spatiotemporal SVD allows for signal separation between tissue, blood, and random noise components by decomposing raw data into spatiotemporal singular vectors, enabling selection of singular vectors with relevant spatiotemporal fluctuations.^29^ SVD to improve image reconstruction performance for photoacoustic computed tomography systems (PACT) has been reported.^32^^,^^33^ For example, Wang et al.^33^ proposed a fast spatiotemporal image reconstruction algorithm with SVD for dynamic PACT and reported accuracy improvement over conventional approaches. In this paper, however, we focus on improving the quality of PA images collected using linear array US transducers. For linear array PAI, SVD has been used for identification and reduction of laser-induced noise using the spatial singular value spectrum (SVS).^34^ Spatiotemporal clutter filtering with SVD has also been applied for contrast-enhanced PAI in a phantom study.^35^ However, the spatiotemporal variation of PA signals was not investigated previously in the context of improving the quality of DAS beamformed label-free murine cardiac PAI data except in our previous conference publication.^26^ The novelty of our approach is to utilize the natural deformation of myocardial tissue to achieve PA image enhancement using spatiotemporal SVD processing. The purpose of this study is to demonstrate PA signal enhancement in myocardial tissue when compared to surrounding muscle tissue and blood within the LV chamber.

Briefly, a custom ECG-R gating algorithm along with a DAS and minimum variance (MV) beamformer is used to reconstruct a cardiac cycle of PAI data. We hypothesize that blood signals from the LV chamber will have low spatiotemporal coherence when compared to signals from the myocardial wall and surrounding tissue region appearing as random temporally incoherent clutter signals. Moreover, as the myocardium contracts and relaxes during a cardiac cycle, myocardial echo signals will have lower spatiotemporal coherence when compared to quasi-static surrounding tissue and any diffuse quasi-static clutter. Based on the aforementioned hypotheses, spatiotemporal SVD processing was applied to enhance the contribution from myocardial tissue.

The paper reports on two main contributions. First, spatiotemporal SVD processing using ECG and respiratory signal gated *in vivo* cardiac murine PAI data acquired using linear array-based PA system is described and implemented. Second, a detailed *in vivo* feasibility study is performed using eight healthy mice along with rigorous quantitative evaluation in terms of contrast ratio (CR), generalized contrast-to-ratio (gCNR), and SNR metrics.

## 2 Materials and Methods

**Fig. 1:**
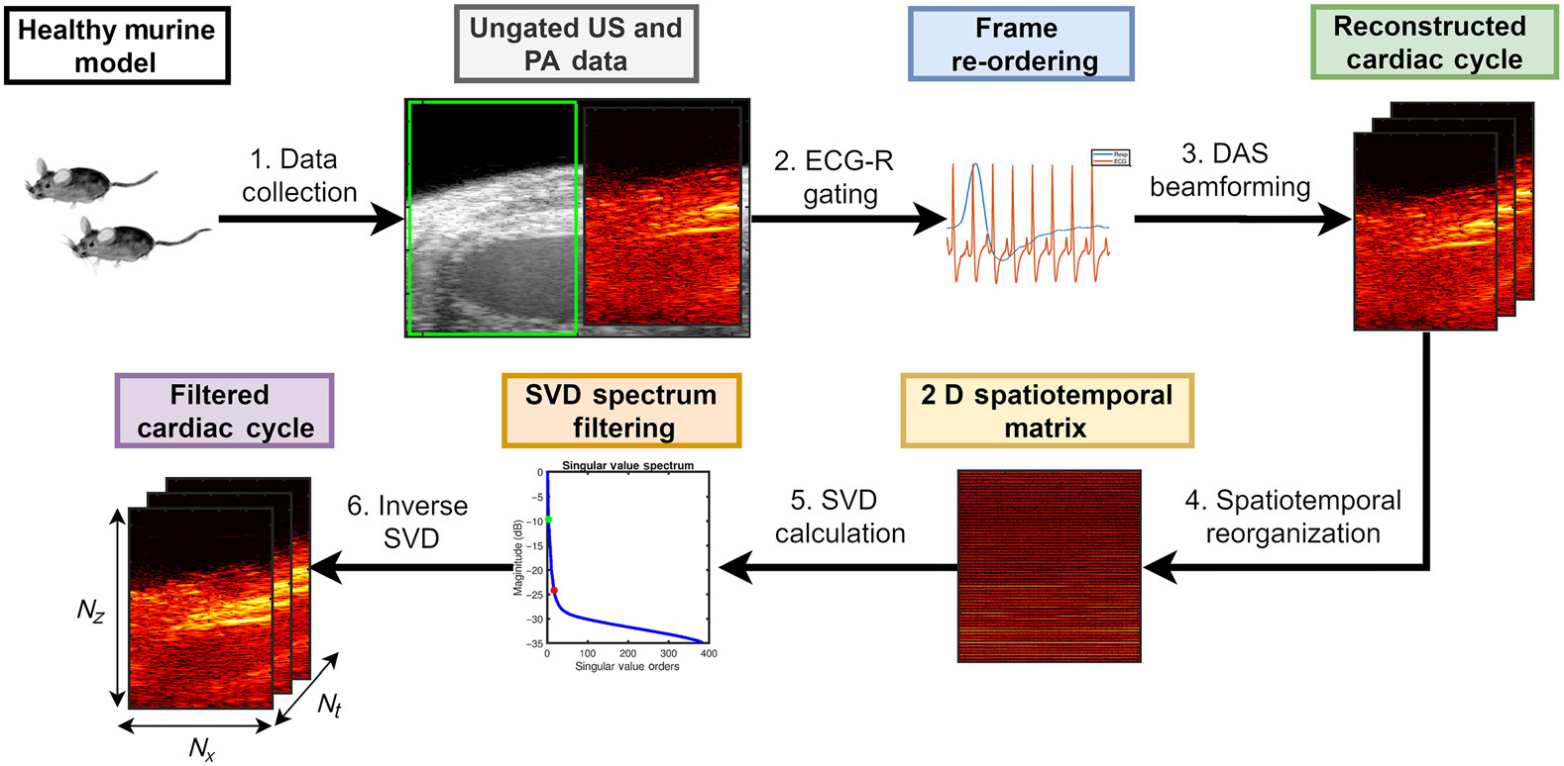
Schematic diagram illustrating the spatiotemporal SVD processing algorithm for ECG and respiratory (ECG-R) gated *in vivo* cardiac PAI.

Figure 1 shows a schematic diagram describing the spatiotemporal SVD algorithm for ECG-R gated *in vivo* cardiac PAI, which is described in detail below.

### 2.1 *In Vivo* Murine Cardiac PAI Data Acquisition

Eight healthy BALB/CJ mice (median age of 10 weeks, five males, and three females) acquired from the Jackson Laboratory (Bar Harbor, Maine) were used to perform an *in vivo* validation study for the proposed SVD processing framework. All *in vivo* experiments were approved by the Institutional Animal Care and Use Committee at the University of Wisconsin–Madison. A Vevo 2100-LAZR PA-ultrasonic imaging system (FUJIFILM VisualSonics, Inc., Toronto, Canada) was utilized for collecting PAI data. After removing chest hair with depilatory cream, Nair (Church & Dwight Co., Ewing, New Jersey), mice were placed in the supine position on a heated platform under anesthesia (1.5% to 3.5% isoflurane) and continuous flow of oxygen (1 to 2  l/min) via a nose cone. ECG and respiratory signals were collected using dedicated physiological monitoring system with the Vevo 2100-LAZR. Spectra 360 electrode gel (Parker Labs, Fairfield, New Jersey) was applied on the physiological signal monitoring system electrodes to ensure optimal contact with each paw ensuring high-quality ECG and respiratory signal acquisition. The supply of isoflurane and oxygen flow rate was titrated to maintain a consistent heart rate between 310 to 340 beats per minute (bpm) as best as possible during imaging.

A LZ 250 transducer (256-element linear array) having a pitch of 90  μm, center frequency of 21 MHz, and bandwidth from 13 to 24 MHz was used for data collection.^36^ LithoClear, (Next Medical Products, Branchburg, New Jersey) a high viscosity acoustic gel, was applied within the cup of the transducer along with a liberal amount to the animals’ chest to ensure optimal acoustic coupling between the transducer and mice while also allowing for a gel offset to reduce reverberation artifacts. Acoustic gel was centrifuged prior to imaging to remove air bubbles that would cause artifacts in PAI. Parasternal long-axis imaging view was used with US B-mode imaging. B-mode images had a depth of 16 mm and width of 11.04 mm with a depth offset of 5 mm and focus at 11 mm. The skin surface of the mice was placed at an approximate depth of 8 mm whenever possible to avoid reverberation artifacts from the skin.^11^^,^^37^ A cine loop of US B-mode was collected to confirm normal cardiac function for each mouse. Then, 1000 frames of co-registered beamformed US and pre-beamformed PA channel data were acquired using an optical wavelength of 850 nm where oxygenated hemoglobin has dominant absorption^38^ with simultaneous acquisition of ECG and respiratory signals. With the LZ 250, two sequential laser pulses are required to cover the chosen US imaging width (11.04 mm) with 64-element parallel acquisition per pulse resulting in a PAI frame rate of one half the laser repetition rate.^39^ To perform PA imaging at the maximum laser repetition rate of the system dedicated Nd:YAG laser (20 Hz), PA imaging width was adjusted to be approximately half of the US imaging width resulting in an acquisition with only 64-elements (green rectangle in Fig. 1).^17^^,^^40^ No frame or A-line averaging was performed during PA data collection. PA gain (40 dB) and time gain compensation were kept constant throughout the experiment to allow inter-animal comparison. Finally, in-phase and quadrature (IQ) sampled PA channel data were exported for offline beamforming and SVD processing.

### 2.2 Cardiac Cycle Reconstruction using ECG-R Gating and Beamforming

A cardiac cycle of PA channel data was reconstructed by performing respiratory signal gating to discard frames and avoid motion artifacts, followed by re-ordering of gated frames using ECG signals and individual frame time stamps. To ensure accurate respiratory signal gating, a publicly available open-source respiratory signal processing toolbox named BreathMetrics (https://github.com/zelanolab/breathmetrics) was used.^41^ Respiratory signal was analyzed to determine all inhalation peak time points with corresponding inhalation onsets and exhalation pause onsets. Then, gating was done per inhalation peak with gate start and end time corresponding to the inhalation onset and exhalation pause onset times, respectively. Any PA and US frames within the gated region were discarded from subsequent analysis. Finally, the remaining usable frames were re-ordered by calculating the delay between the image time stamps and nearest ECG R-waves reconstructing a cardiac cycle of US and PA channel data. Additionally, an ECG curve for the gated cardiac cycle PA data was reconstructed using the image time stamps of the re-ordered frames after ECG-R gating and the original ECG timing information. To reconstruct the gated ECG curve, we sampled the original ECG signal by finding time indices closest to the image time stamps of the re-ordered usable frames after performing ECG-R gating. The reconstructed ECG curves are presented in the video files in Sec. 3 (Figs. 5 and 7).

PA complex radio-frequency IQ data were reconstructed from PA channel data using DAS beamforming with 64-element aperture, uniform aperture weighting, and dynamic apodization with f-number of 1. Dynamic receive focusing was performed by calculating one-way US signal propagation delay assuming the speed of sound to be $1540\;{\mathrm{ms}}^{-1}$. Beamforming process was accelerated by implementation using CUDA to run on a GPU in MATLAB (Mathworks Inc., Massachusetts). All beamforming was done on an Intel(R) Xeon(R) CPU E5-2640 v4 at 2.40 GHz and a Tesla K40c GPU (compute capability 3.5). This resulted in a three-dimensional (3D) complex-valued matrix P used for SVD processing with dimensions $N_{x}=64$ A-lines, $N_{z}=296$ samples along depth, and Nt≈300 to 400 frames.

Additionally, time-delayed PA channel data were also beamformed using an MV beamforming algorithm.^42^ For MV, the optimal aperture apodization function was determined by minimizing the variance of beamformed data using the following equation:

$$W_{\mathrm{MV}}(t)=\frac{R_{\mathrm{SA}}{\left(t\right)}^{-1}a}{a^{H}R_{\mathrm{SA}}{\left(t\right)}^{-1}a},$$ (1)

where $W_{\mathrm{MV}}(t)$ is the minimum variance aperture weighting vector, a (the steering vector) is a unit vector in our case due to dynamic receive focusing, $R_{\mathrm{SA}}(t)$ is the co-variance matrix estimated by dividing the full array into overlapping sub-arrays having a length of $N_{s}=16$, and t is the time-of-arrival of PA acoustic waves. MV beamforming was accelerated using the Parallel Computing Toolbox in MATLAB.

### 2.3 Spatiotemporal Singular Value Decomposition Processing

Theoretical background on spatiotemporal SVD processing is presented in this section. For SVD processing, a 3D complex-valued matrix P is constructed using stacks of ECG-R gated DAS beamformed PAI cardiac cycle data. The matrix P has two dimensions in space denoted by $N_{x}$ and $N_{z}$ corresponding to the number of transducer elements and number of samples along the depth axis respectively and one-dimension (1D) in time ($N_{t}$) corresponding to the number of frames in the ECG-R gated cardiac cycle data. A spatiotemporal reorganization was applied on the matrix P to construct a two-dimensional (2D) Casorati matrix, S with dimensions of ($N_{x}\times N_{z}$) by $N_{t}$.^29^ Each column vector of S represents a PA image. Then, SVD is performed on S, which can be represented as follows:

$$S={U\Delta V}^{*},$$ (2)

where Δ is a diagonal matrix with dimensions [$\min(N_{x}\times N_{z},N_{t})$ by $\min(N_{x}\times N_{z},N_{t})$] containing the singular values in the diagonal and two unitary matrices U with dimensions [($N_{x}\times N_{z}$) by $\min(N_{x}\times N_{z},N_{t})$] and V dimensions [$N_{t}$ by $\min(N_{x}\times N_{z},N_{t})$] containing the spatial and temporal singular vectors corresponding to each singular value, respectively.

**Fig. 2:**
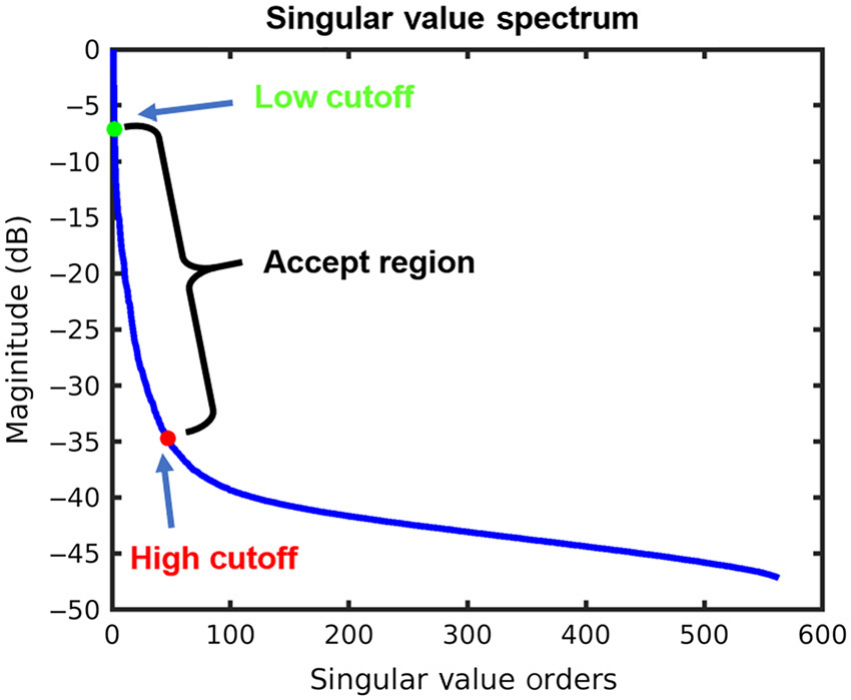
SVS derived from SVD of *in vivo* cardiac PAI murine data. Green and red dots show the low and high-order cutoff respectively for SVD filtering.

For cardiac PAI, we are interested in enhancing signals from myocardial tissue depicting natural contraction and relaxation over a cardiac cycle. The key assumption here is that myocardial tissue should have lower spatiotemporal coherence compared to PA signals from diffuse quasi-static clutter and surrounding muscle regions and higher spatiotemporal coherence compared to fast-moving blood volumes inside the LV chamber. The assumed spatiotemporal PA signal fluctuation will be characterized by matrix V containing the temporal singular vectors. Therefore, to enhance myocardial PA signals, singular values and vectors associated with myocardial tissue displacements were preserved by filtering both lower and higher-order singular values of the SVS. The low-order cutoff used to separate myocardial PA signal from quasi-static clutter and surrounding muscle was manually selected and denoted as $r_{\mathrm{st}}$ here and in the rest of the paper. After application of ECG-R gating, we observed that high amplitude PA signals from the surrounding muscle regions were depicted as quasi-static clutter while myocardial PA signals had deformation characteristics associated with natural contraction and relaxation of the heart over a cardiac cycle. Spatiotemporal SVD decomposed the raw PA data into spatiotemporal singular vectors. The singular vectors from quasi-static clutter and surrounding muscle had the lowest spatiotemporal fluctuations thereby contributing to lower-order singular values. On the other hand, myocardial tissue had higher spatiotemporal fluctuations, therefore, utilizing a lower-order cutoff enhanced the myocardial PA signals over quasi-static clutter and surrounding muscle. The high-order cutoff used to suppress random PA noise was calculated using the gradient of SVS and selected at the singular value order where gradient becomes <20 and denoted by $r_{\mathrm{rt}}$. The filtered SVS can be presented using a truncated diagonal matrix $\Delta^{ST}$ as follows:

$$\Delta^{ST}=\Delta \times I^{ST}$$ (3)

where $I^{ST}$ is a diagonal matrix to filter Δ. For $I^{ST}$, diagonal elements between $r_{\mathrm{st}}$ and $r_{\mathrm{rt}}$ were set to one and rest were set to zeros. A typical SVS derived from our cardiac PAI data with chosen low- and high-order cutoff is shown in Fig. 2. A filtered Casorati matrix, $S^{ST}$ through inverse SVD calculation was derived using the following equation.

$$S^{ST}={U\Delta }^{ST}V^{*}.$$ (4)

Finally, a 3D matrix of SVD processed cardiac PAI data, $P^{ST}$ was reconstructed by applying a spatiotemporal reorganization on $S^{ST}$.

### 2.4 Quantitative Analysis

**Fig. 3:**
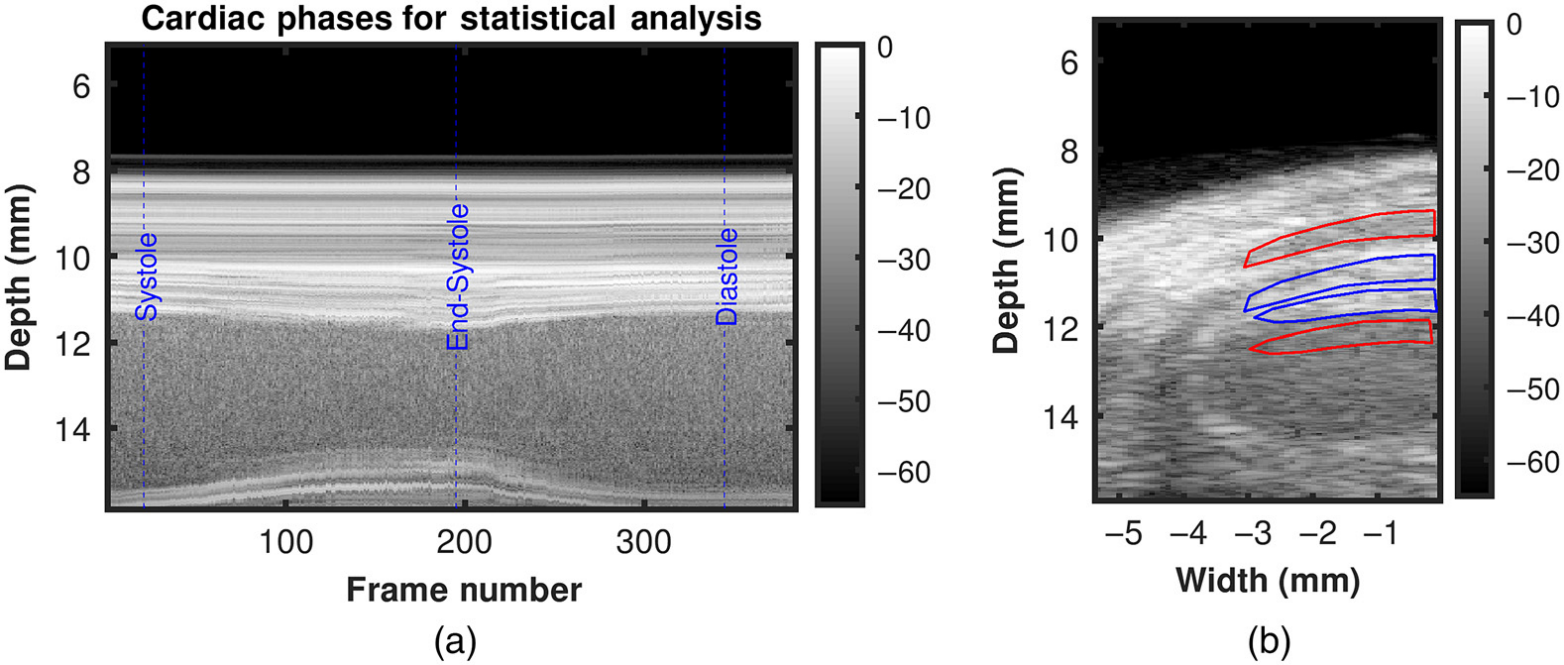
US guided statistical analysis of *in vivo* PAI. (a) US M-mode image derived using the reconstructed cardiac cycle after ECG-R gating. Chosen cardiac phases are shown with blue dashed line on the M-mode image. (b) Representative target (blue polygon) and background (red polygon) ROIs overlayed on PAI co-registered US image.

To perform quantitative analysis, three cardiac time points (during systole, at end-systole and during diastole) were identified using US M-Mode image derived from the reconstructed ECG-R gated cardiac cycle of the co-registered US B-mode cine-loop [Fig. 3(a)]. We define systole as the cardiac phase when the LV chamber begins to contract until just before it reaches it smallest dimension, end-systole as the cardiac time point at which LV chamber is at the smallest dimension, and diastole as the cardiac phase when the LV chamber begins to expand until it reaches it largest dimension. It is worth noting that imaging FOV was set to focus on the interventricular septum while maintaining enough offset between skin and transducer face to avoid reverberation artifact during PAI. Then, corresponding B-mode images were used to manually draw target and background region of interest (ROI) as shown with blue and red polygons in Fig. 3(b), respectively. Both target and background ROI had equal area. Finally, corresponding DAS, MV, and SVD processed PA images were evaluated by calculating CR,^17^^,^^40^ gCNR,^43^^,^^44^ and SNR^45^ using the following equations:

$$CR=20\times \log_{10}(\frac{\mu_{t}}{\mu_{b}}),$$ (5)

$$gCNR=1-\overset{N-1}{\underset{l=0}{\sum }}\min\{k_{t}(x_{l}),k_{b}(x_{l})\},$$ (6)

$$SNR=20\times \log_{10}(\frac{\mu_{t}}{\sigma_{b}}),$$ (7)

where $\mu_{t}$ and $\mu_{b}$ denote the average envelope detected PA signal amplitudes for target and background ROI, respectively. In Eq. (3), $k_{t}$ and $k_{b}$ represent the target and background histograms, respectively, calculated by dividing the entire range of PAI values into 100 bins (N) with bin centers denoted by l.

For statistical analysis, one-way analysis of variance (ANOVA) with the Bonferroni multiple comparison test was used to compare among DAS, MV and SVD-4. It is worth noting that SVD-4 denotes spatiotemporal SVD processed image with $r_{\mathrm{st}}=4$. Statistical analysis and graphing were done with Origin, Version 2020 (OriginLab Corporation, Northampton, Massachusetts).

## 3 Results

**Fig. 4:**
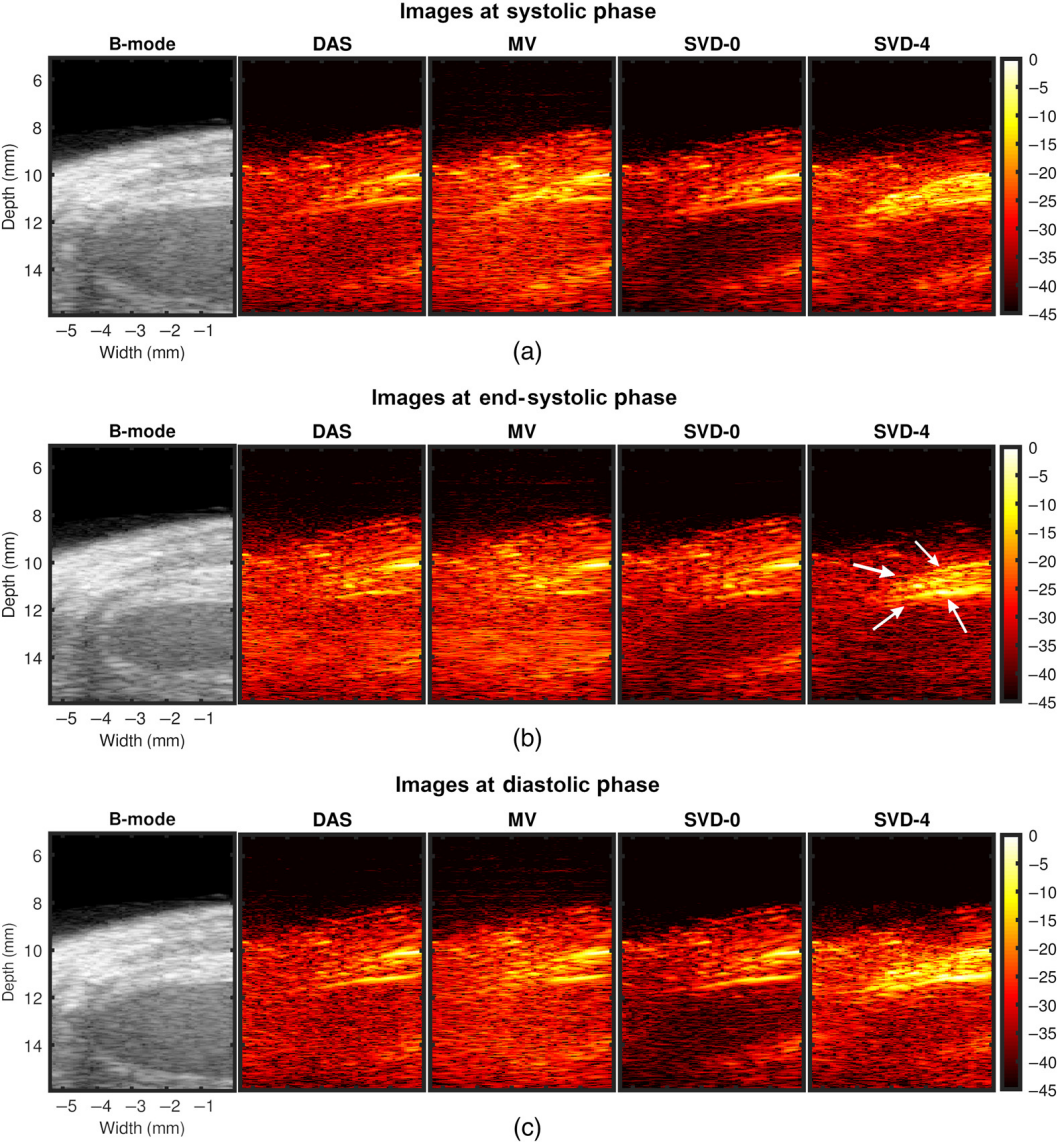
Representative SVD processed images at three different cardiac time points demonstrating improved PAI signal specificity after processing. Panels (a)–(c) show results at systolic, end-systolic, and diastolic phase of a cardiac cycle, respectively. US B-mode and PA images for DAS, MV, SVD-0, and SVD-4 are presented from left to right chronologically for each sub-figure. SVD-0 and SVD-4 denote spatiotemporal SVD processed images with $r_{\mathrm{st}}=0$ and 4, respectively.

Figures 4(a)–4(c) show representative examples of DAS, MV, and SVD processed images during systole, at end systole (ES), and during diastole of a cardiac cycle, respectively. US B-mode and PA images reconstructed with DAS, MV, SVD-0, and SVD-4 are presented from left to right chronologically for each sub-figure. PA signal strength from the myocardium in DAS and MV results were low making myocardial signal localization difficult. With SVD-0, no significant qualitative difference was observed in the myocardial wall region. However, significant myocardial PA signal enhancement was achieved with SVD-4. Specifically, we observe ES radial wall thickening in the SVD-4 image, which was not clear in DAS, MV, and SVD-0 results [Fig. 4(b)]. The radial wall thickening was confirmed with the corresponding US B-mode image [Fig. 4(b) leftmost image].

**Fig. 5:**
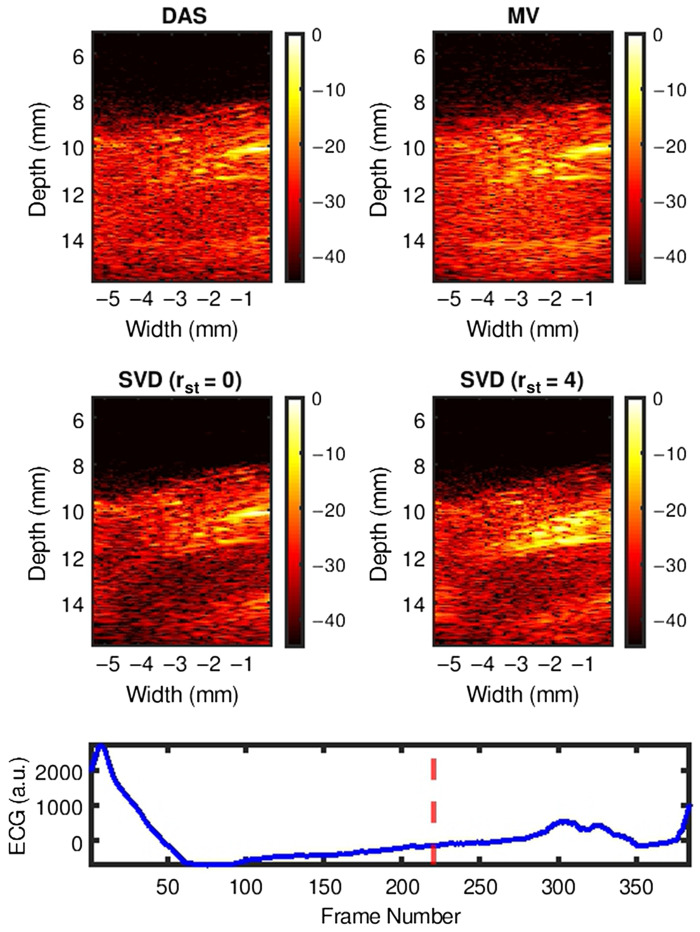
Video of SVD processed cardiac cine-loop demonstrating improved PAI signal specificity after processing (Video 1, MP4, 12.115 MB [URL: https://doi.org/10.1117/1.JBO.26.4.046001.1]).

Figure 5 shows the SVD processed cardiac cine-loop comparison with DAS and MV beamformed cardiac cine-loop. SVD-0 does not improve myocardial signal specificity when compared to DAS and MV however, note the reduced temporal variation of the noise background. On the other hand, SVD-4 demonstrates significant improvement in both myocardial signal specificity with reduced temporal variation of noise background and show that SVD processing preserves underlying cardiac motion.

**Fig. 6:**
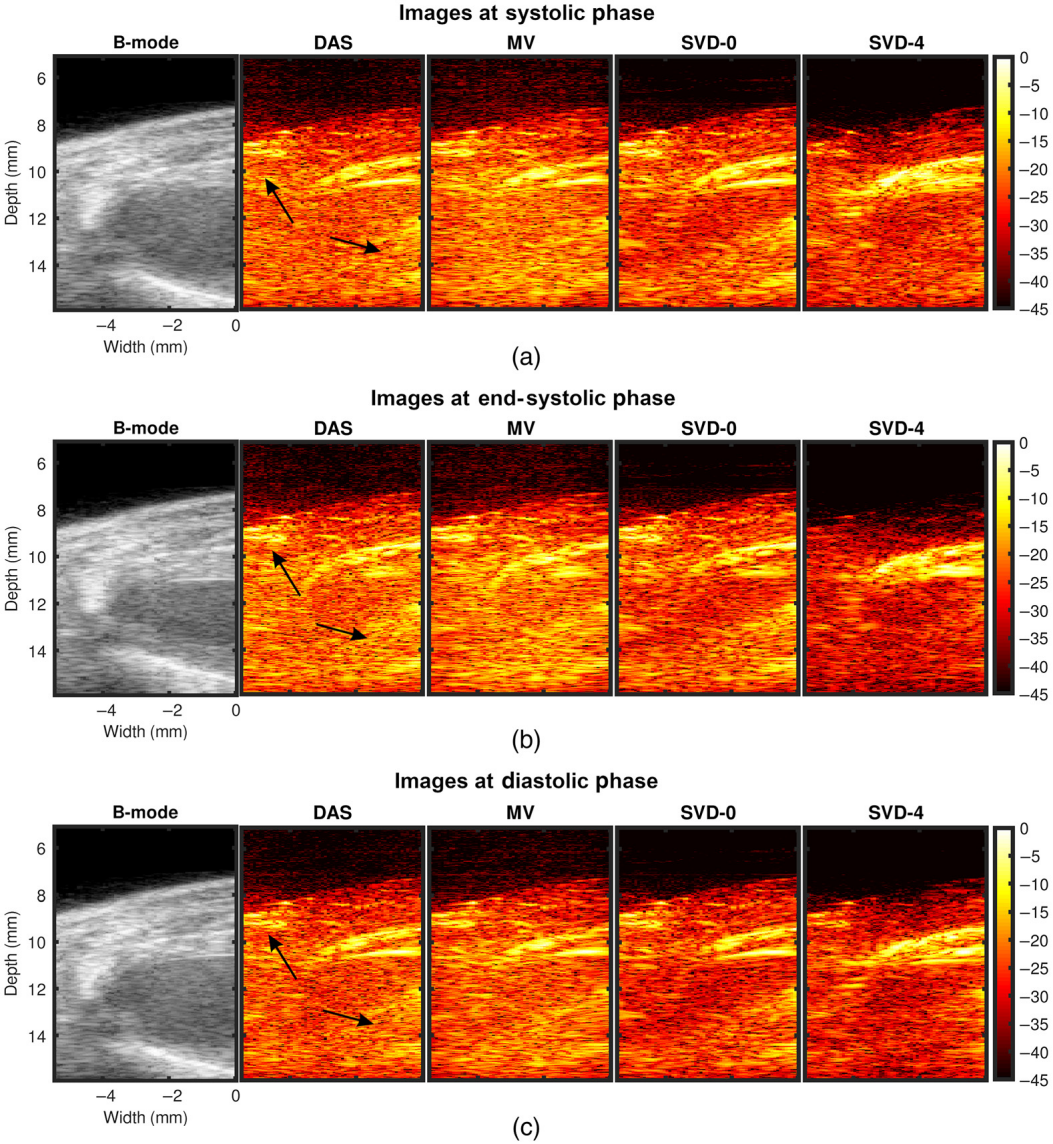
Representative SVD processed images at three different cardiac time points demonstrating PAI diffuse and quasi-static clutter reduction after processing. Panels (a)–(c) show results at systolic, end-systolic, and diastolic phase of a cardiac cycle, respectively. US B-mode and PA images for DAS, MV, SVD-0, and SVD-4 are presented from left to right chronologically for each sub-figure. SVD-0 and SVD-4 denote spatiotemporal SVD processed images with $r_{\mathrm{st}}=0$ and 4, respectively.

Figures 6(a)–6(c) show another set of representative examples of DAS, MV, and SVD processed images during systole, at ES and during diastole of a cardiac cycle, respectively. US B-mode and PA images reconstructed with DAS, MV, SVD-0, and SVD-4 are presented from left to right chronologically for each sub-figure. In DAS and MV results, spurious high amplitude PA clutter (diffuse quasi-static) signals are observed in the surrounding muscle and background regions (indicated using black arrows in Fig. 6 DAS images). Though some level of clutter reduction was observed with SVD-0, high amplitude PA signals persist in the regions indicated with arrows in DAS results. Finally, with SVD-4 significant PAI diffuse quasi-static clutter reduction was achieved compared to DAS, MV, and SVD-0 thus enhancing signal specificity and detectability of myocardial PA signals.

**Fig. 7:**
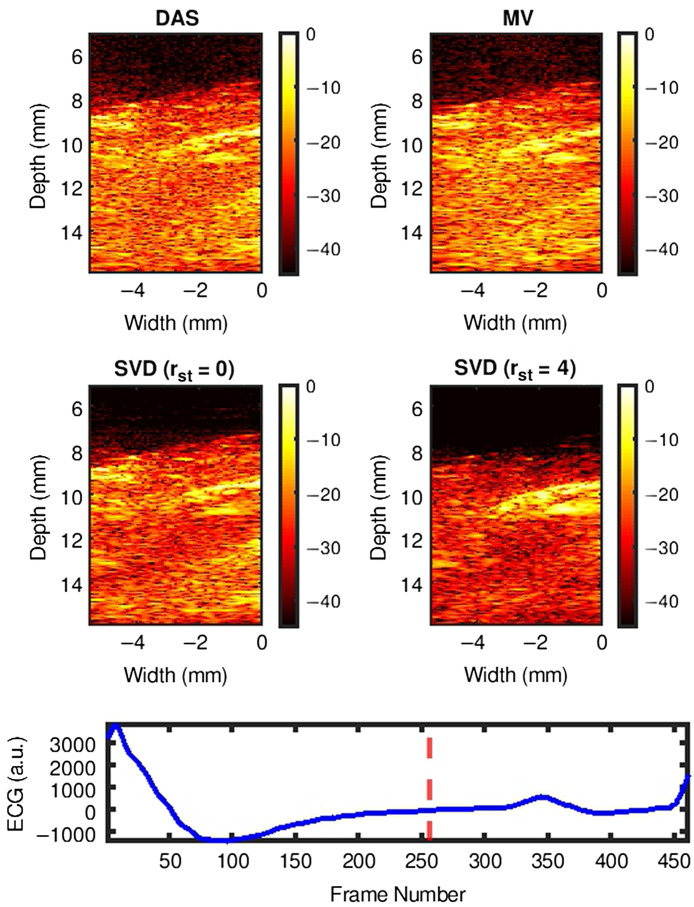
Video of SVD processed cardiac cine-loop demonstrating PAI diffuse and quasi-static clutter reduction after processing (Video 2, MP4, 12 MB [URL: https://doi.org/10.1117/1.JBO.26.4.046001.2]).).

Figure 7 shows the SVD processed cardiac cine-loop comparison with DAS and MV beamformed cardiac cine-loop for Fig. 6. Note that with SVD-0 we see reduced temporal variation in the noise background but diffuse quasi-static clutter signals persist. However, SVD-4 demonstrate marked reduction in the diffuse quasi-static clutter signals along with reduced temporal variation of noise background and preserved underlying cardiac motion.

**Fig. 8:**
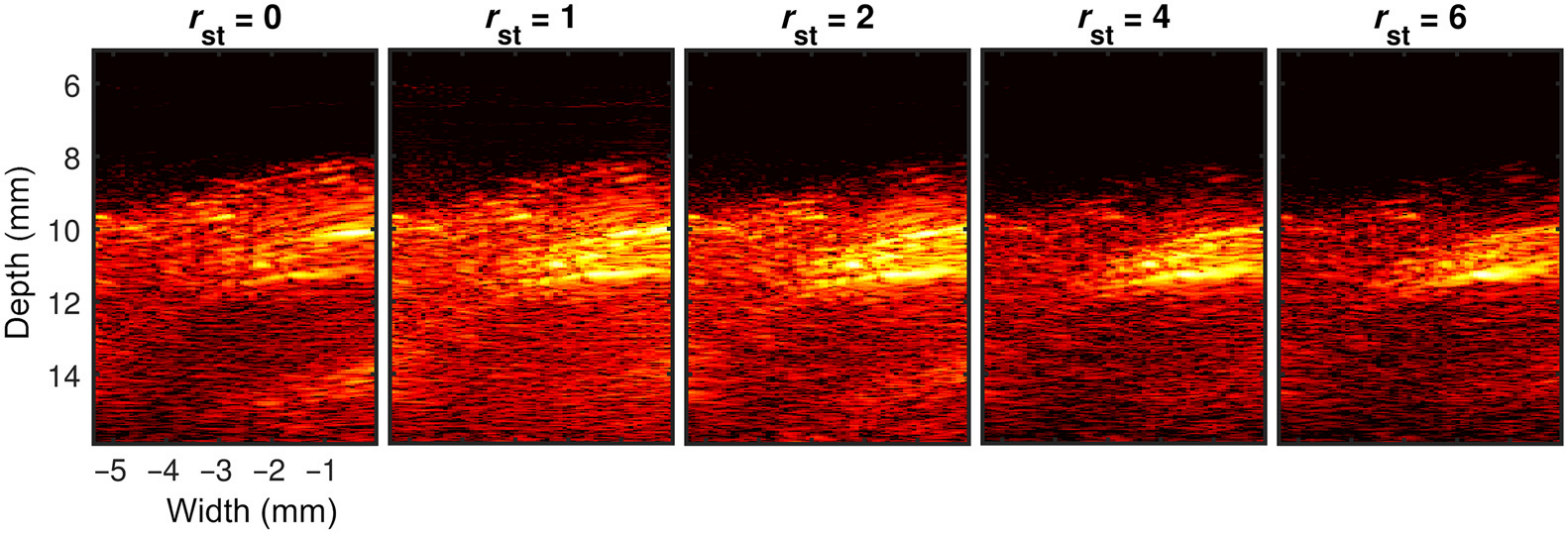
End-systole spatiotemporal SVD processed images as a function of lower singular valuer order cutoff threshold ($r_{\mathrm{st}}$). Results with $r_{\mathrm{st}}=0$, 1, 2, 4, and 6 are presented from left to right chronologically.

**Fig. 9:**
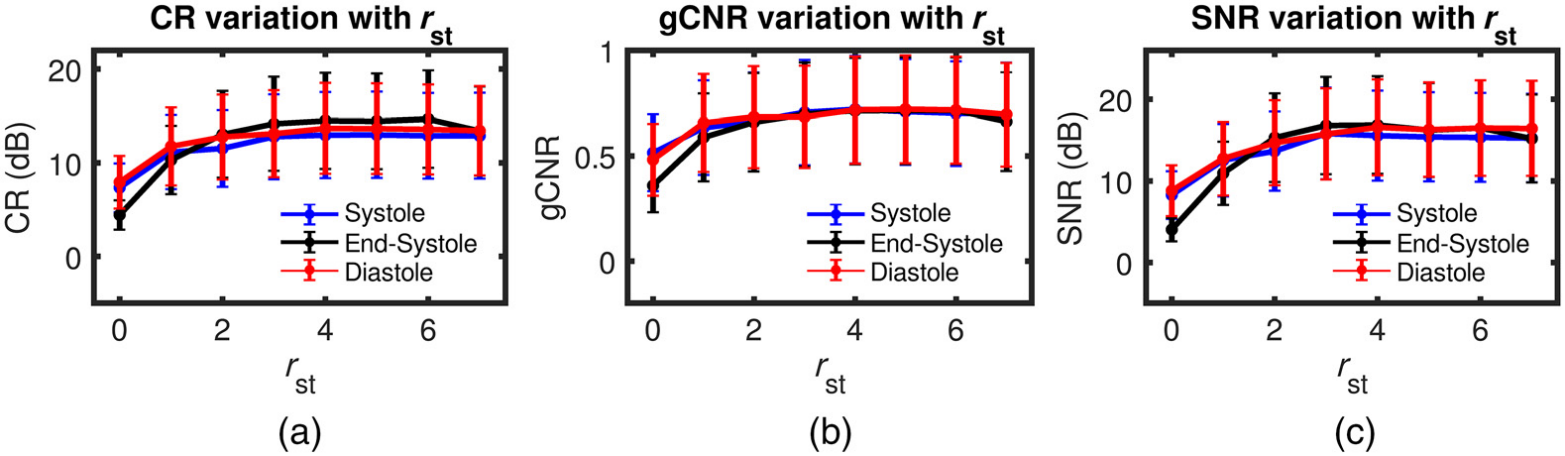
Variation of (a) CR, (b) gCNR, and (c) SNR as a function of $r_{\mathrm{st}}$ for spatiotemporal SVD processed images evaluated at systolic (blue), end-systolic (black), and diastolic (red) phase of a cardiac cycle.

Findings from a parametric study to investigate the performance of the proposed algorithm as a function of lower-order singular value cutoff ($r_{\mathrm{st}}$) are summarized in Figs. 8 and 9. Representative end-systole spatiotemporal SVD processed images for different $r_{\mathrm{st}}$ values are presented in Fig. 8. Results with $r_{\mathrm{st}}=0$, 1, 2, 4, and 6 are presented from left to right chronologically. The impact of the $r_{\mathrm{st}}$ cutoff is evident in these results in terms of myocardial signal enhancement and background signal suppression, with the best quality image obtained at $r_{\mathrm{st}}=4$. However, choosing too high $r_{\mathrm{st}}$ may suppress signals from myocardial tissue as seen in Fig. 8 for $r_{\mathrm{st}}=6$. Figures 9(a)–9(c) show the variation of CR, gCNR, and SNR as a function of $r_{\mathrm{st}}$ for systolic, end-systolic, and diastolic phase SVD processed PA images, respectively. We observe peak CR, gCNR, and SNR were achieved with $r_{\mathrm{st}}=4$ after which the curves plateau. Therefore, SVD processed image with $r_{\mathrm{st}}=4$ was used in the quantitative comparative study against DAS and MV beamforming.

**Fig. 10:**
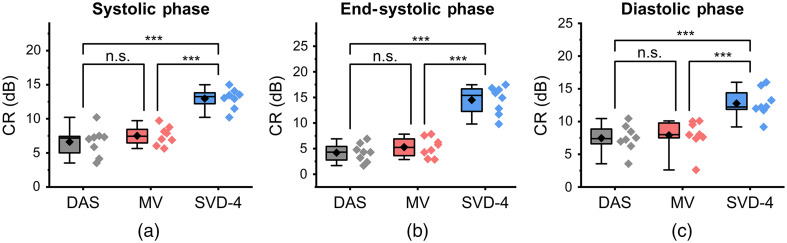
Statistical analysis for CR comparison among DAS, MV, and SVD-4 (n=8). Panels (a)–(c) show results at systolic, end-systolic, and diastolic phase of a cardiac cycle, respectively. SVD-4 presents with statistically higher CR values when compared to DAS and MV.

**Fig. 11:**
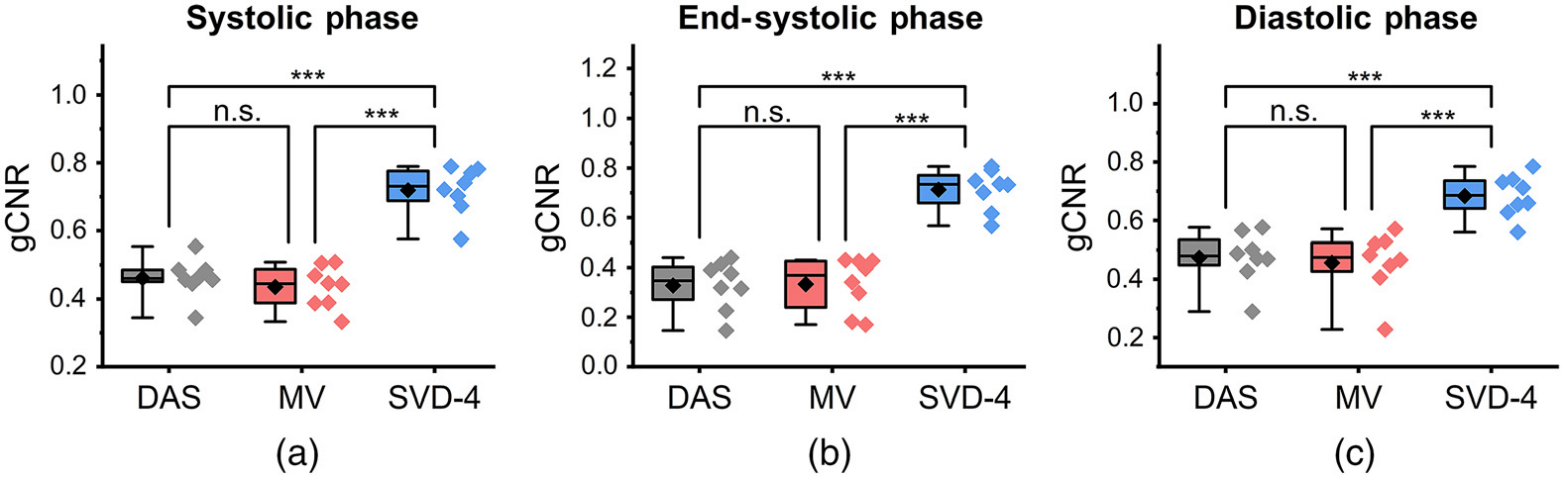
Statistical analysis for gCNR comparison among DAS, MV, and SVD-4 (n=8). Panels (a)–(c) show results at systolic, end-systolic, and diastolic phase of a cardiac cycle, respectively. SVD-4 shows statistically higher gCNR values when compared to DAS and MV.

**Fig. 12:**
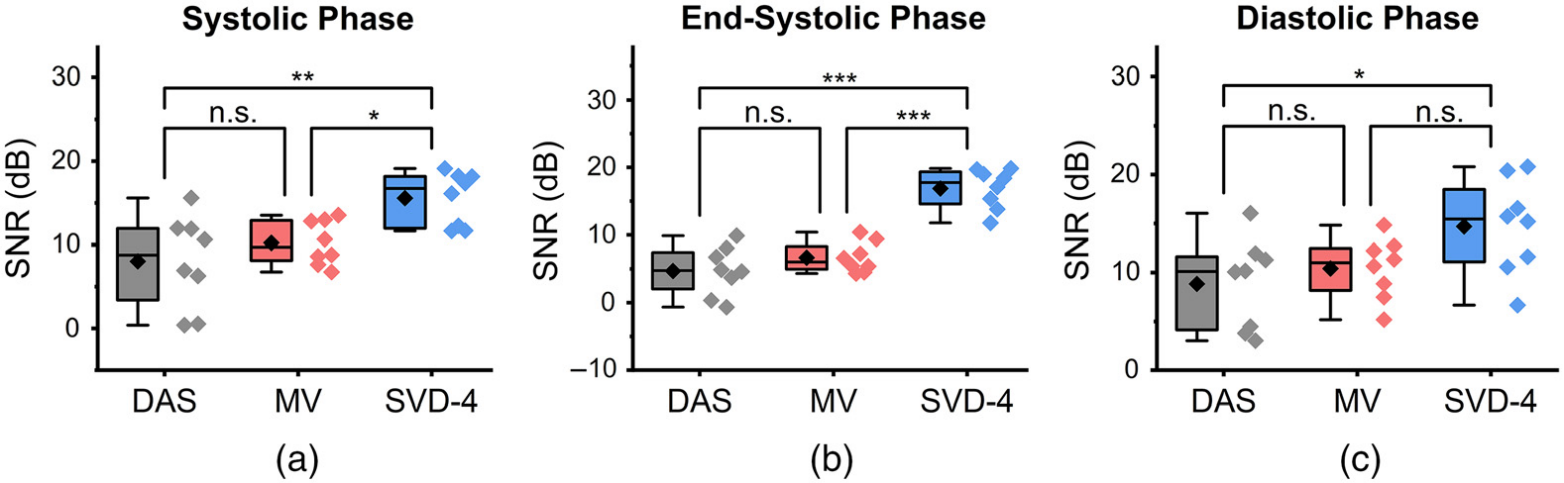
Statistical analysis for SNR comparison among DAS, MV and SVD-4 (n=8). Panels (a)–(c) show results at systolic, end-systolic, and diastolic phase of a cardiac cycle, respectively. SVD-4 had statistically higher SNR values than DAS.

Quantitative comparison results using CR, gCNR, and SNR are summarized in Figs. 10–12, respectively. Results are presented using box-whisker plots with raw data plotted on the right side. Mean of each distribution is illustrated by the black diamond symbol.

Figures 10(a)–10(c) show the CR results during systolic, at end-systolic, and during the diastolic phase of a cardiac cycle, respectively. SVD-4 had higher CR values compared to DAS and MV with statistical significance for all cases. No statistically significant differences were observed between DAS and MV. For example, at ES, mean CR values for DAS, MV, and SVD-4 were 4.20, 5.28, and 14.49 dB, respectively.

Figures 11(a)–11(c) show the gCNR results during systolic, end-systolic, and during the diastolic phase of a cardiac cycle, respectively. SVD-4 had higher gCNR values compared to DAS and MV with statistical significance for all cases. No statistically significant difference was observed between DAS and MV. Larger differences were observed in the ES phase when compared to systolic and diastolic phases. For example, at ES, mean gCNR values for SVD-4 were 115.15% higher than DAS, whereas at systolic phase, it was 56.52% higher.

Figures 12(a)–12(c) show the SNR results during systolic, at end-systolic, and during the diastolic phase of a cardiac cycle, respectively. For all three phases, SVD-4 had statistically higher SNR than DAS. When compared to MV, SVD-4 had statistically higher SNR at ES and systole with no statistically significant difference during the diastolic phase. However, highest mean SNR values were achieved in all three phases using SVD-4. For example, mean SNR of DAS, MV, and SVD-4 were 8.84, 10.41, and 14.69 dB for the diastolic phase results.

Table 1 summarizes the computation times required to reconstruct a PA cardiac cycle using DAS, MV, and spatiotemporal SVD processing for two mice. For example, DAS requires 45.81 s to reconstruct a complete 3D cardiac cycle having a dimension of 296×64×300 samples while MV requires significantly more time (446.58 s). It is worth noting that enhanced PAI with spatiotemporal SVD can be achieved with a very low additional computation burden (1.71 s). Similar performance trends were observed for mouse 2 with computational time scaled by $N_{t}$ (461 frames).

**Table 1:** Summery of computational times (seconds).

	DAS	MV	SVD^a^
	Total (per frame)	Total (per frame)	
Mouse 1	45.81 (0.12)	446.58 (1.16)	1.71
Mouse 2	54.40 (0.12)	506.61 (1.10)	2.45

Note: Mouse 1, $N_{t}=300$ frames, Mouse 2, $N_{t}=461$ frames. DAS = Delay-and-sum, MV= minimum variance, SVD = singular value decomposition

a Additional time needed to process entire cardiac cycle using spatiotemporal SVD after DAS.

## 4 Discussion

In this paper, a spatiotemporal SVD algorithm with ECG and respiratory (ECG-R) gating for *in vivo* cardiac PAI has been proposed and validated. *In vivo* feasibility with eight healthy mice demonstrated significantly improved performance with SVD-4 processing over conventional DAS and MV beamformed images. The proposed SVD processing is a data-driven approach where spatiotemporal characteristics of cardiac PAI are utilized to enhance signal contribution from myocardial tissue under the following assumptions based on literature findings and experimental observations. First, highly absorbing blood inside the coronary artery (murine arterial oxygen saturation ≈90% to 95%^11^^,^^16^) having low blood flow velocity (diastolic coronary flow velocity ≈20  cm/s^46^) should contribute to the PA signals from myocardial tissue at 850 nm. Second, highly scattering mice skin and muscle due to the presence of connective tissues and anisotropic layers of collagen,^47^ having lower optical absorption coefficients at 850 nm (for example, male BALB/CJ mice skin optical absorption coefficient at $850\;\mathrm{nm}\approx 1\;{\mathrm{cm}}^{-1}$^47^) compared to oxygenated blood, should result in low amplitude PA signals compared to myocardial tissue. During data collection, we observed the presence of spurious high amplitude PA clutter signals from surrounding muscle, which were quasi-static in nature. Third, PA transients from the large volume of high velocity circulating blood (in early filling, E wave and late or atrial filling phase, A wave during diastole) inside the LV generates mainly destructive interference during DAS beamforming, resulting in non-viable PA signals with random spatiotemporal fluctuations. It is worth noting that the E and A wave velocity^48^ of mitral valve flow during diastole have previously been reported to be ∼54.2 and 43.8  cm/s, respectively.^49^ Furthermore, short-duration pulses provided to the flash lamp within the laser source may also contribute to random PA noise.^34^ Therefore, in the proposed method, singular values and vectors corresponding to cardiac tissue displacements associated with the natural contraction and relaxation of the heart over a cardiac cycle were preserved by discarding the first few singular values for the low-order SVD cutoff to suppress spurious high amplitude quasi-static clutter and by suppressing random PA signal fluctuations using high-order SVD cutoff (Fig. 2). To ensure that a suitable dataset is generated for SVD processing, a custom ECG-R gating algorithm was developed using an open-source Matlab toolbox.

Qualitative results shown in Figs. 4–7 show that significant improvement in myocardial signal specificity is achieved with $r_{\mathrm{st}}=4$, which was also validated by quantitative analysis. It is worth noting that no additional temporal smoothing was applied to preserve the original spatial and temporal resolution demonstrating a significant improvement over prior approaches using higher persistence (Figs. 5 and 7).^11^^,^^37^ With SVD processing, significant enhancement of myocardial signal was demonstrated with improved contrast between the myocardium and background as demonstrated by CR comparison results (Fig. 10). Additionally, gCNR comparison was done to confirm that this contrast enhancement was not due to mere dynamic range alternation rather target detectability. gCNR results shown in Figure 11 show that myocardial signal detectability is significantly improved using spatiotemporal SVD processing when compared to conventional DAS or MV results. Higher gCNR improvement observed at ES compared to either systolic or diastolic phases can be attributed to the high strain rate at ES with the thickest wall dimension.^50^ SNR results demonstrate statistically significant improvement with SVD-4 over DAS for all cardiac phases. We observed an exception in the diastolic phase where MV and SVD-4 had non-significant differences. In contrast to CR and gCNR (both measure target detectability), SNR additionally considers the smoothness of the background regions. To understand the SNR trend, we also evaluated the mean PA amplitude of the target region and standard deviation of background region individually and found that SVD-4 had higher mean PA amplitudes demonstrating improved myocardial signal enhancement in all phases corroborating the improvement in CR and gCNR. However, reduction in background standard deviation in diastolic phase was not as significant as in the end-systole and systolic phase resulting in non-significant SNR improvement statistically between MV and SVD images even though SVD-4 had higher mean SNR value. Overall, qualitative and quantitative results demonstrate that spatiotemporal SVD processing can potentially improve *in vivo* cardiac PAI quality.

**Fig. 13:**
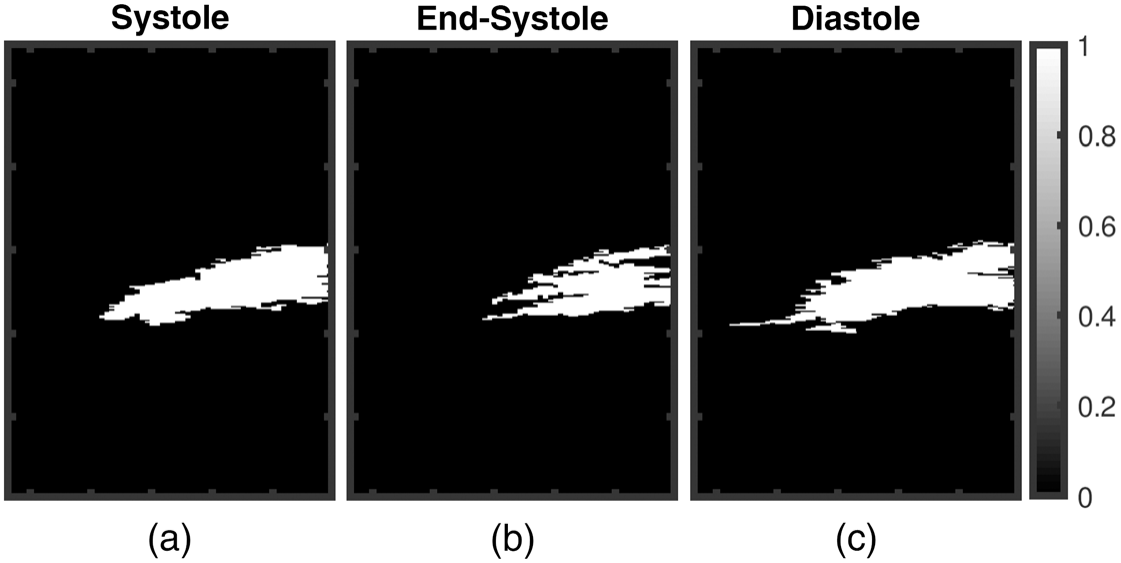
(a)–(c) Binary maps of the myocardial wall generated by applying a threshold on SVD-4 images at systolic, end-systolic, and diastolic phases of cardiac cycle, respectively. Radial thickening and longitudinal shortening of the wall at ES is observed in Fig. 13(b).

It is worth noting that myocardial tissue identified in SVD processed PA images showed similar anatomical variation as a function of time as observed in B-mode images. For example, in Fig. 4(b), thickening and shortening of the anterior wall is evident from the B-mode image at ES. Observe thickening and shortening of the anterior wall from SVD-4 images [myocardial boundaries indicated with arrows in Fig. 4(b)] with clear contrast when compared to the background. Binary maps were generated by applying a threshold on the SVD-4 images (from Fig. 4) at the systolic, end-systolic, and diastolic phases, which are shown in Fig. 13. Anatomical variation at different cardiac phases can be clearly observed in Fig. 13, demonstrating that both spatial and temporal localization of myocardial PA signals is achieved using spatiotemporal SVD processing. One common approach in PA-based oxygen saturation (% ${\mathrm{sO}}_{2}$) estimation is to use a quality control region-of-interest (ROI).^38^^,^^51^ In the future, we will utilize the SVD processed images to define our quality control ROI utilizing improved target detectability and perform multispectral imaging to evaluate the myocardial % ${\mathrm{sO}}_{2}$ as a function of time over a cardiac cycle.

The performance of SVD processing depends on the choice of lower singular valuer order cutoff threshold ($r_{\mathrm{st}}$), which was chosen empirically by evaluating a range of $r_{\mathrm{st}}$ values [Figs. 8 and 9]. However, this is not an optimal solution when the proposed algorithm must be applied to larger datasets. In future work, we will investigate the feasibility for automated determination of the low order cutoff threshold by estimating the mean frequency of each temporal singular vectors contained in the matrix V.^30^

In this paper, the focus was on *in vivo* murine cardiac PAI where the myocardial signals are diffuse in nature. PAI has also been used for imaging prostate brachytherapy seeds,^52^^,^^53^ percutaneous radiofrequency ablation needle detection,^54^ and surgical guidance^7^ where the signals of interest appear to be more coherent. We anticipate that our proposed spatiotemporal SVD processing can be applied for those applications with appropriate adjustment of singular value thresholds. Adaptive beamforming methods such as MV, DMAS beamforming^22^ can be also be coupled with SVD processing to improve murine cardiac PAI quality if channel data is accessible. However, researchers must be mindful of any non-linearity introduced by these adaptive beamforming algorithms.

Despite the presented encouraging results, this study still has some limitations. First, the SVD processing was considered as decomposing the matrix S into weighted, ordered sum of separable matrices as hypothesized for ultrafast functional US imaging.^29^^,^^30^ However, from our study we observed some overlap between the myocardial and background signal subspace even after applying SVS thresholding. Therefore, to account for the background signal, further signal processing approaches are necessary. One potential approach might be the use of photoacoustic sub-aperture processing (PSAP) developed by our lab to suppress incoherent clutter for DAS PA images.^55^ One example with PSAP processing to suppress background signals in the SVD processed images is presented in Appendix A. Second, the low and higher order singular value cutoffs were chosen empirically and were fixed for all mice. However, it is anticipated that adaptive methods^30^^,^^31^ for selecting the singular value cutoff may improve performance further by accounting for physiological variation (for example, heart rate under anesthesia) that occurs with different mice. Third, any singular value below low order and above high order singular value cutoff was set to zero in our implementation. However, adaptive weighting functions based on the singular values^27^ can be can be designed to weight the SVS to further enhance myocardial PA signals. Fourth, only healthy murine model was considered in this study. However, efficacy should be evaluated for murine cardiovascular disease models such ischemia-reperfusion^13^ for further validation, which will be studied in future.

## 5 Conclusion

In this work, a spatiotemporal SVD method for *in vivo* murine cardiac PAI data was demonstrated. Qualitative and quantitative comparison between conventional DAS, MV, and SVD processing show that higher quality single wavelength *in vivo* cardiac PA images can be realized using the proposed method.

## 6 Appendix A: Background Suppression Using PSAP

**Fig. 14:**
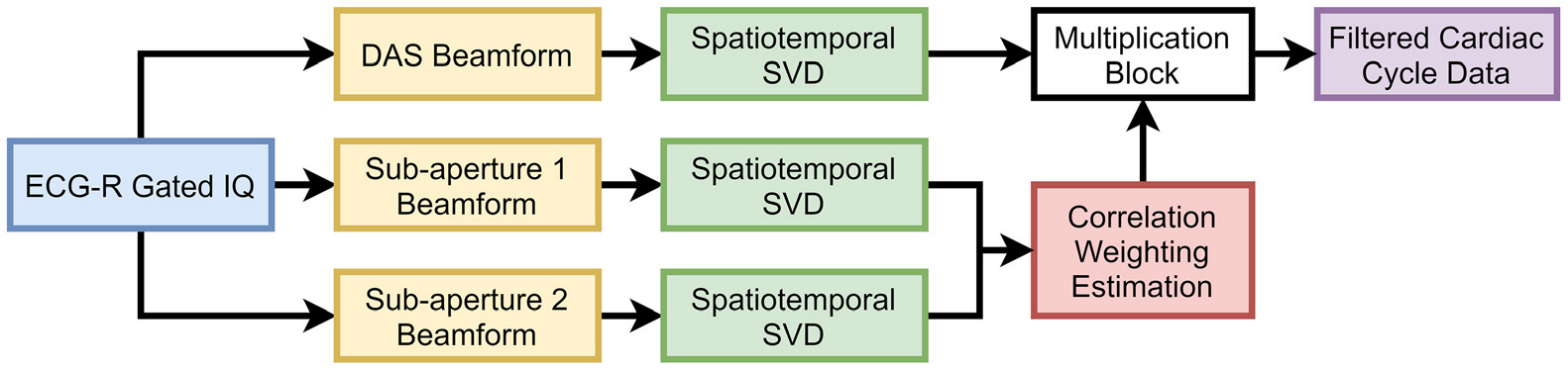
Schematic diagram demonstrating coupled PSAP and SVD processing.

Figure 14 presents a schematic diagram demonstrating coupled PSAP and SVD processing for background suppression in the DAS SVD processed images. In addition to DAS beamforming with full aperture, beamforming was also done with two non-overlapping sub-apertures having no common elements defined using binary weighting vectors. Here, sub-aperture 1 ($S_{1}$) weighting vector was constructed of ones and zeros with an alternating pattern of two elements and sub-aperture 2 ($S_{2}$) weighting vector was complementary of sub-aperture 1. Further details on PSAP can be found in Ref. 55. Both cardiac cycle data reconstructed with $S_{1}$ and $S_{2}$ were filtered with the proposed spatiotemporal SVD method (Sec. 2.1). Then, 3D (2D space + 1D time) weighting matrix ($W_{\mathrm{PSAP}}$) was determined by calculating zero lag normalized cross-correlation (NCC) between each frame of $S_{1}$ and $S_{2}$ reconstructed cardiac cycle. During NCC calculation, incoherent clutter signals from background have low similarity while myocardial PA signal have high similarity.^55^ Therefore, DAS SVD processed images were multiplied with $W_{\mathrm{PSAP}}$ to suppress background signals. The resultant images are denoted as PSAP-SVD.

**Fig. 15:**
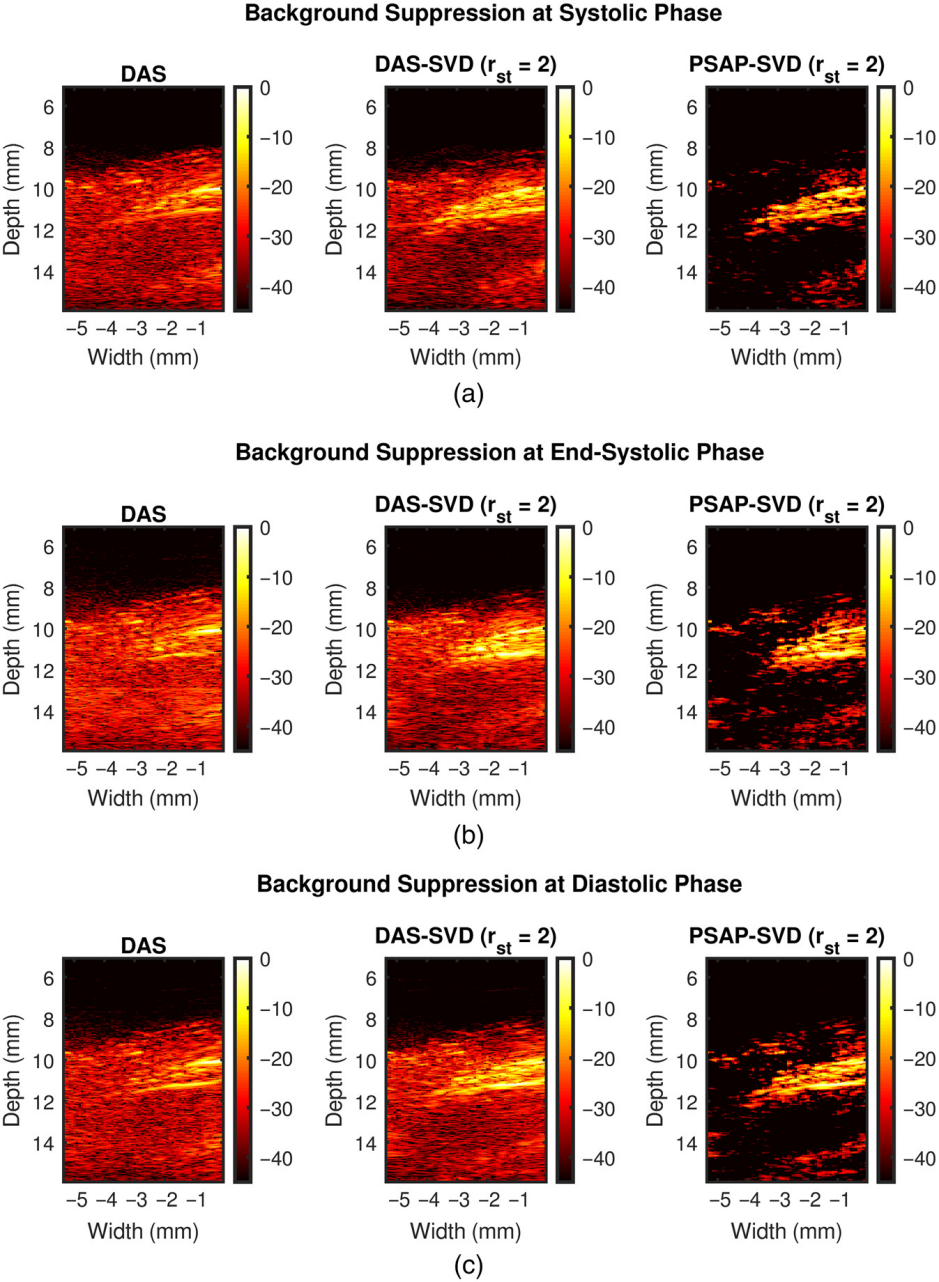
Representative background suppression results from coupled PSAP and SVD processing. (a)–(c) show results at systolic, end-systolic, and diastolic phase of a cardiac cycle, respectively. Results with DAS, DAS-SVD ($r_{\mathrm{st}}=2$), and PSAP-SVD ($r_{\mathrm{st}}=2$) are presented from left to right chronologically for each sub-figure. $r_{\mathrm{st}}$ denotes the lower singular value order chosen for thresholding.

Figure 15 shows representative background suppression results from coupled PSAP and SVD processing. Figures 15(a)–15(c) show results at systolic, end-systolic, and diastolic phase of a cardiac cycle, respectively. Results with DAS, DAS-SVD ($r_{\mathrm{st}}=2$), and PSAP-SVD ($r_{\mathrm{st}}=2$) are presented from left to right chronologically for each sub-figure. We observe that coupled PSAP and SVD processing achieved simultaneous suppression of background signal and enhancement of myocardial PA signal for all three cases.

## Code, Data, and Materials Availability

Associated codes are made available on github: https://github.com/Mukaddim/Spatiotemporal-SVD-Cardiac-PAI.git.
